# Power Bars: Mormon Crickets Get Immunity Boost from Eating Grasshoppers

**DOI:** 10.3390/insects14110868

**Published:** 2023-11-10

**Authors:** Robert B. Srygley, David H. Branson

**Affiliations:** Pest Management Research Unit, Northern Plains Agricultural Research Laboratory, USDA-Agricultural Research Service, 1500 N. Central Ave., Sidney, MT 59270, USA; dave.branson@usda.gov

**Keywords:** katydid, nutrition, omnivory, Orthoptera, predator, prey, rangeland, Wyoming

## Abstract

**Simple Summary:**

Mormon crickets (*Anabrus simplex)* are omnivorous, feeding on plants, fungi, and insects, including one another. Because insects contain more protein than plants, prey availability might determine the protein consumed by Mormon crickets. Some grasshoppers co-occur with Mormon crickets and feed on the same hostplants, but little is known about their interactions. We hypothesized that if Mormon crickets feed on grasshoppers, then the Mormon crickets’ needs for alternative protein sources would decline when grasshoppers were more numerous. In addition, because Mormon crickets with less dietary protein had less immunity, we hypothesized that greater grasshopper density would enhance Mormon cricket immunity. In a field setting, we varied the numbers of Mormon crickets from 0 to 20 and the numbers of grasshoppers *Melanoplus borealis* from 0 to 45 m^−2^ in 68 1-m^2^ cages. After one month, we measured Mormon cricket dietary preferences and immune activity. As predicted, we found that protein consumption from the alternative source declined as grasshopper density increased, and immunocompetence increased with grasshopper availability. In addition, plant nitrogen declined with increasing insect density, reinforcing the importance of predation by Mormon crickets to meet their protein needs. Potentially influencing management decisions, Mormon crickets affect grasshopper populations, and grasshopper abundance might be an indicator of Mormon cricket immunity.

**Abstract:**

In addition to feeding on plants, Mormon crickets *Anabrus simplex* Haldeman, 1852 predate on invertebrates, including one another, which effectively drives their migration. Carnivory derives from lack of dietary protein, with Mormon crickets deprived of protein having less phenoloxidase (PO) available to combat foreign invaders, such as fungal pathogens. Because Mormon crickets commonly occur with grasshoppers that feed on the same plants, we investigated interactions between grasshoppers and Mormon crickets, and hypothesized that if Mormon crickets are predatory on grasshoppers, grasshopper abundance would influence the protein available to Mormon crickets and their immunity. In a field setting, we varied densities of Mormon crickets (0, 10, or 20 per cage) and grasshoppers *Melanoplus borealis* (0, 15, 30, or 45) in 68 1-m^2^ cages. After one month, we measured Mormon cricket dietary preferences and PO activity. As predicted, artificial diet consumption shifted away from protein as grasshopper density increased, and immunocompetence, as measured by PO activity, also increased with grasshopper availability. Although nitrogen availability in the vegetation decreased with increasing insect density, predation became an important source of protein for Mormon crickets that enhanced immunity. Grasshoppers can be an important source of dietary protein for Mormon crickets, with prey availability affecting Mormon cricket immunity to diseases.

## 1. Introduction

Omnivores consume a broad array of animals and plants. In terrestrial environments, plants, which are relatively dilute in protein, are much more common than the more protein-rich animals, such that variability in animal prey encounters can result in large individual differences in dietary intake. Many phenotypic traits that directly impact fitness, such as growth [1], immunity [2], body size, aggression [3], strength [4], and locomotor performance [5], vary with dietary protein. Omnivorous animals as diverse as primates and bears, ants and Mormon crickets show variation in these traits in association with diet. Hence, dietary protein is an important constituent of an omnivorous diet. If capable of encountering and capturing animal prey, omnivores will obtain greater concentrations of dietary protein from these than from plants.

The omnivorous Mormon cricket *Anabrus simplex* Haldeman, 1852 (Orthoptera: Tettigoniidae) is well known for its carnivory. In addition to plants and fungi, Mormon crickets consume invertebrate prey and even cannibalize one another [6,7]. Seeking protein, cannibalistic Mormon crickets can effectively drive the migration of conspecifics [8]. Flightless, Mormon crickets march in dense, uninterrupted bands 1–2 km across [6,9]. Studies of Mormon crickets and migratory locusts both indicate that coordinated, unidirectional movement of conspecifics minimizes the chances of being encountered and cannibalized when the insects achieve sufficient densities [10,11].

Migrating Mormon crickets that lack protein in their diet are also deficient in phenoloxidase (PO) activity [12], an enzyme involved in the insects’ generalized immune response to wounding and invasion. Supplementing the diets of migrating Mormon crickets with protein resulted in increased PO activity and decreased locomotor activity [12]. In captive Mormon crickets, restricting dietary protein reduces PO activity and makes the insects more susceptible to fungal attack [13].

If the principal source of protein for Mormon crickets is invertebrate prey, then prey availability should be an important factor determining PO activity. In nature, Mormon crickets commonly occur in the same habitat as grasshoppers (Orthoptera: Acrididae) that feed on the same plants and can reach densities that make them the most common insect herbivore on rangeland [14]. As a result, they are likely to frequently encounter one another in nature, and yet, interactions between grasshoppers and Mormon crickets have not been explored. Several characteristics suggest that Mormon crickets not only compete for the same host plants, but predate directly on grasshoppers. First, Mormon crickets hatch earlier in the spring than grasshopper species that do not overwinter as nymphs or adults, and so they tend to be more developmentally advanced than most grasshopper species. Second, adults are larger (1–9 g) than most grasshopper species, and so, Mormon crickets have a size advantage in contests. Third, the earlier spring hatching suggests that Mormon crickets might be active at cooler temperatures than many grasshopper species, and this could permit Mormon crickets to seek and capture grasshoppers at times, such as early in the morning, when the potential prey are unable to bask effectively to elevate their body temperatures. In this paper, we investigated interactions between Mormon crickets *Anabrus simplex* and the Northern grasshopper *Melanoplus borealis* to test the hypothesis that grasshopper abundance in a Wyoming mountain meadow increases Mormon cricket enzymatic-based immunity to infectious diseases.

## 2. Materials and Methods

### 2.1. Study Site, Study Organisms, and Cage Trials

The study site was a mountain meadow located on Forest Service Road (FSR) 167 (44°48′27.049″ N, 107°32′32.809″ W, 2405 m) in the Bighorn Mountains near Burgess Junction, WY, USA where Mormon crickets and Northern grasshoppers regularly occur in high densities. The meadow serves as summer pasture for cattle with a variety of grasses and broad-leaved forbs, such as silky lupine *Lupinus sericeus*, sticky geranium *Geranium viscosissimum*, and mountain yarrow *Achillea lanulosa* [15]. We constructed 68 1 m^2^ cages made of Lumite insect netting (1 m^2^ × 0.7 m in height, SI Corporation, Gainesville, Georgia) with 15 cm polyester flaps at the base, which laid flat around the exterior of the cage. Each cage was pulled onto a PVC pipe frame after clearing the 1 m^2^ area of insects and spiders, and the flaps were fastened to the ground with spikes driven through grommet holes and weighted down with sandbags to seal the cage [16]. Researchers accessed the interior through zippers on two sides. Northern grasshoppers were collected within 1 km of the site. They were sorted at Burgess Junction to remove those younger than 3rd instars, and then placed into cages at densities of 0, 15, 30, and 45 grasshoppers m^−2^. Initially, only grasshoppers were stocked so that any immediate losses were not due to Mormon crickets. We counted the grasshoppers one week after the initial stocking restocked each cage with a sufficient number of grasshoppers to return to the treatment density for each cage, and then introduced the prescribed density of Mormon crickets. Mormon crickets were collected from the same area and from a meadow on FSR 17 (Paint Rock Road, 44°27′48.853″ N, 107°27′38.279″ W, 2655 m), which is at approximately the same elevation as the study site on FSR 167. An equal number of male and female Mormon crickets were added to the cages at combined densities of 0, 10, and 20 m^−2^. A total of 48 cages held four replicates of each density combination. At stocking, Mormon crickets were in the final three immature stages (5th, 6th, or 7th instar). In order to allow for the removal of Mormon crickets to evaluate their immunity and diet intake without disturbing the 48 cages described above, we stocked 20 additional cages: 8 cages with 10 male and 10 female Mormon crickets and either 0, 15, 30, or 45 northern grasshoppers m^−2^ (two replicates of each combination), and 12 cages with 5 male and 5 female Mormon crickets and either 0, 15, 30, or 45 northern grasshoppers (three replicates of each combination). In these 20 cages, we maximized the effects of the grasshoppers on the Mormon cricket immunity and diet by combining the two species from the outset. One week after the initial stocking, we counted the grasshoppers and restocked each cage to its prescribed density. Although Mormon cricket presence in each cage was verified, they were not restocked.

### 2.2. Immunity Assays

Approximately one month from the date when the cages were stocked, we drew hemolymph by puncturing the arthrodial membrane at the base of each insect’s hindleg with a 26-gauge hypodermic needle. We aimed to collect hemolymph from male and female Mormon crickets in each density treatment. However, we collected hemolymph of 27 insects (Table 1) because meadow voles and pocket gophers had compromised the integrity of most of the cages, allowing Mormon crickets to escape through the holes dug in the soil. Hemolymph from the wound was collected in a 20 µL capillary, and 8 µL was diluted 1:50 with cold phosphate buffered saline (PBS) solution to be used in assays of spontaneous phenoloxidase (PO) and prophenoloxidase (proPO) enzymatic activity and total hemolymph protein.

To assay spontaneous PO activity, we followed the protocols detailed in [12]. Briefly, samples of thawed hemolymph diluted in PBS were centrifuged and activated with 10 mM dopamine solution. The plate was loaded into a temperature-controlled Biotek microplate reader (25° C), and absorbance at 492 nm was read between 5 and 15 min. If sample absorbance was linearly related with time, we calculated mean V (change in absorbance min^−1^). One unit PO activity per mL hemolymph is defined as the amount of enzyme resulting in a 0.001 increase in absorbance. Prophenoloxidase (proPO), a zymogen of PO, is activated by chymotrypsin. To measure total PO activity (both PO and proPO), we dissolved 1 mg alpha-chymotrypsin from bovine pancreas (Sigma, St. Louis, MO, USA) in 1 mL PBS, combined an equal volume of this solution with centrifuged hemolymph in PBS (1:50), and incubated for 30 min. In the plate wells, we added 5 µL of the incubated solution to 195 µL 10 mM dopamine. As for spontaneous PO, mean V was calculated from plate readings between 5–15 min to measure total PO activity in units mL^−1^ hemolymph. We measured total hemolymph protein in mg protein mL^−1^ hemolymph with a Total Protein Kit, Micro (Sigma) compared to a serial dilution of the human albumin standard.

### 2.3. Intake Diets

Approximately one month from the date when cages were stocked, we characterized the intake diets of Mormon crickets in each density treatment (10 or 20 Mormon crickets and 0, 15, 30, or 45 grasshoppers m^−2^) following the methods of [8]. We prepared a 42% protein diet consisting of a 3:1:1 mix of casein, peptone, and albumen and a 42% carbohydrate diet consisting of equal parts of sucrose and dextrin. Both diets contained 54% cellulose and 1.8% Wesson’s salt mixture and 2.2% vitamins, linoleic acid and cholesterol. In a free-choice experiment, we aimed to collect five male and five female Mormon crickets from each density treatment, but due to the loss of most of the Mormon crickets when cages were compromised, we presented diets to 43 insects (Table 1). The insects were housed individually with free access to water and 0.75 g of each diet for 24 h. After 24 h, the diets were removed and replaced with fresh diet, which remained with the insects for an additional 24 h. Diets were dried and weighed in the lab. The dry masses consumed from each diet is a measure of the relative intake of carbohydrates and protein over the first and second 24 h period. Body mass of each cricket was measured with an Ohaus microbalance. The 10 × 0 male was excluded because a P-diet molded and could not be weighed accurately; also excluded were a male 20 × 30 and a female 10 × 30, which had both died before weighing (Supplementary Table S1).

### 2.4. Vegetation

Near the end of the growing season, approximately two months after the cages were stocked, we clipped vegetation in a 0.25 m^2^ area of each of the ten uncompromised cages, comprising the following treatments one 0 × 0, two 0 × 15, one 0 × 30, two 0 × 45, one 10 × 30, one 20 × 15, one 20 × 30, and one 20 × 45. Above ground vegetation was separated into forbs and grasses, which were dried and weighed separately (Supplementary Table S2). Percent nitrogen and carbon in a 150 mg subsample of each vegetation type from each cage were measured with dry combustion (LECO Corporation, St. Joseph, MI, USA).

### 2.5. Statistical Analyses

Consumption of protein (P) or carbohydrates (C) during each 24 h period (Table S1) was normally distributed following square root transformation (Anderson–Darling Goodness of Fit Test for day 1, square root P, *p* = 0.64; day 1, square root C, *p* = 0.57; day 2, square root P, *p* = 0.54; and day 2, square root C, *p* = 0.11). In a Multivariate Analysis of Variance (MANOVA) for each day separately, P and C were the dependent variables and sex, Mormon cricket density (2-levels), grasshopper density (3-levels), and the Mormon cricket x grasshopper density interaction were the independent variables. For both days, the interaction was not significant (*p* > 0.13) and pooled with error.

Spontaneous PO activity (Table S2) was tested for differences between low and high Mormon cricket densities (10 and 20 m^−2^, respectively), sex, and the two-way interaction with analysis of variance (ANOVA). PO was not significantly different from a normal distribution (*n* = 27, *p* = 0.70). Because of empty cells, we ran a separate ANOVA to test PO activity for differences among grasshopper densities, sex, and the two-way interaction. Mormon crickets housed with medium and high grasshopper densities (30 and 45 m^−2^, respectively) were combined for the analysis because there were only two of the latter. These analyses were repeated for total PO activity, which was normally distributed (*n* = 27, *p* = 0.95), and total hemolymph protein, which was normally distributed following log transformation (*n* = 27, *p* = 0.61).

Biomass of grasses, forbs, and the two combined (Table S3) were not significantly different from normally distributed (*p* > 0.18). Differences in total biomass among Mormon cricket density (three levels) or grasshopper density (four levels) were tested with ANOVA. Percent nitrogen (N) of forbs and grasses was unrelated within a cage (*p* = 0.24), and so, percent N of forbs and percent N of grasses wereassumed to be independent observations. Percent N and the ratio of carbon to nitrogen (C:N) were normally distributed (*p* = 0.46, and *p* = 0.94, respectively). To determine if percent N of the vegetation changed with MC or grasshopper density, we regressed percent N on Mormon cricket and grasshopper densities, separately. We also regressed percent N and C:N on total insect density (Mormon crickets and grasshoppers combined).

## 3. Results

### 3.1. Intake Diets

**Figure 1:**
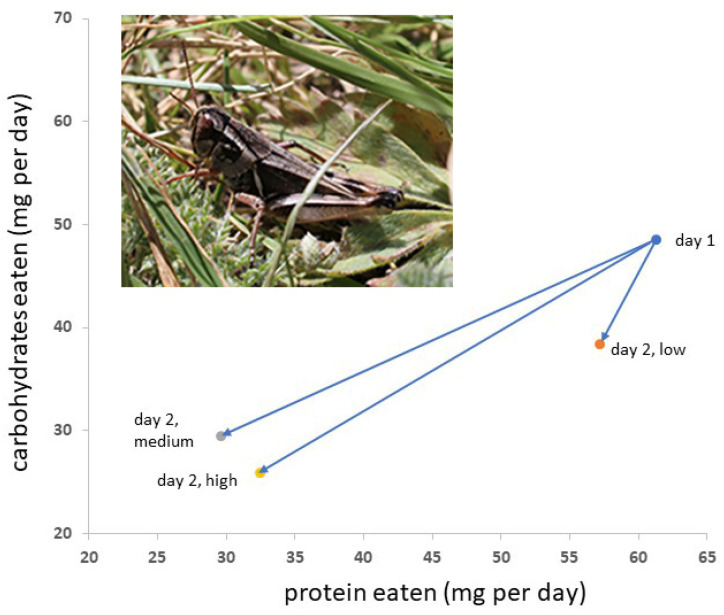
Differences in Mormon cricket diet selection from day 1 to day 2 (arrows) depended on the density of grasshoppers in the insect’s cage. An average value for the two sexes is shown. Grasshopper density had no effect on diet on day 1. Those with medium and high grasshopper densities were not different on day 2, whereas those with low grasshopper densities consumed more protein on day 2. Data were square root-transformed for statistical analyses. Inset: Northern grasshopper adult.

Neither the density of Mormon crickets nor the density of grasshoppers had significant effects on square root-transformed P and C consumption during the first 24 h (Table 2, Figure 1). Sex was a significant factor. For Mormon cricket consumption of square root-transformed P and C during the second 24 h period, sex and grasshopper density were significant factors (Table 2). Univariate tests indicated that P consumption was significantly different between the sexes on day 1 (*p* = 0.0179), but C consumption did not differ significantly (*p* = 0.0998). On day 2, C consumption did not differ significantly between the sexes (*p* = 0.693) or among grasshopper densities (*p* = 0.392), whereas P consumption was significantly different between the sexes (*p* = 0.0031) and grasshopper density (*p* = 0.002). Multiple comparisons of the means indicated that the P consumption of Mormon crickets in a low grasshopper density of 15 m^−2^ was significantly higher than in grasshopper densities of 30 or 45 m^−2^, which did not differ significantly. Body mass did not differ between Mormon cricket stocking densities or grasshopper density (*p* = 0.86 and *p* = 0.40, respectively), but females were significantly larger than males (2.8 g and 2.3 g, respectively, *p* = 0.0090).

### 3.2. Immunity Assays

**Figure 2:**
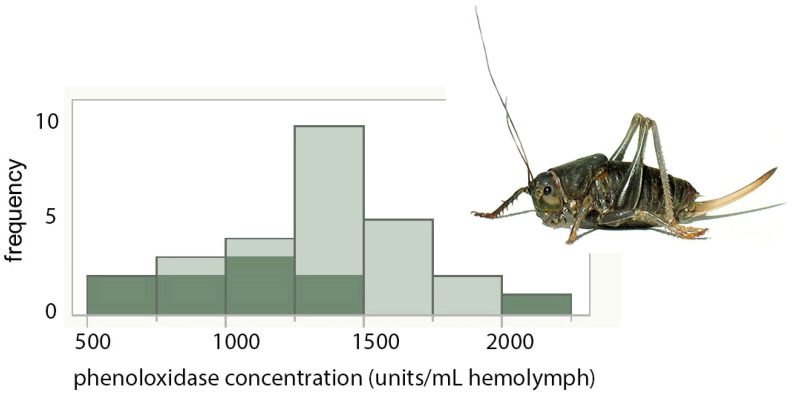
Mormon crickets reared with lower grasshopper densities had significantly lower phenoloxidase titers. Dark shading: low grasshopper density; light shading: medium and high grasshopper densities. Inset: Mormon cricket adult.

The PO titers of Mormon crickets did not vary significantly with sex or Mormon cricket density (*p* > 0.80 and *p* = 0.16, respectively), but did vary significantly with grasshopper density (*p* < 0.0088; low: 1100 ± 102, mean ± s.e.; medium + high: 1465 ± 78 units mL^−1^ hemolymph, Figure 2). Stocking densities of Mormon crickets, grasshoppers, or the sex of the Mormon crickets did not significantly affect total PO titers (PO and proPO combined; *p* = 0.70, *p* = 0.83, and *p* > 0.20, respectively). Log protein was also unaffected by Mormon cricket stocking densities, grasshopper density, or sex (*p* = 0.56, *p* = 0.078, and *p* > 0.35, respectively). Body mass for the insects in this assay did not differ between Mormon cricket stocking densities, grasshopper density, and sex (*p* = 0.24, *p* = 0.38, and *p* = 0.14, respectively).

### 3.3. Vegetation

**Figure 3:**
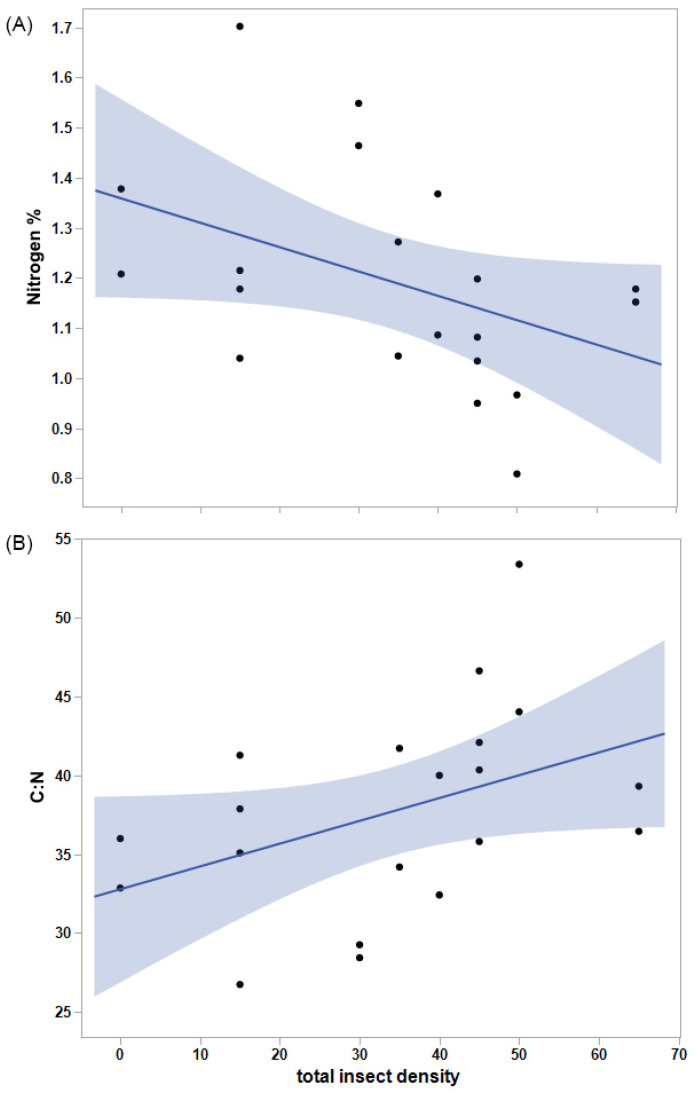
(**A**) Percent nitrogen of vegetation tended to decline with increasing insect density (m^−2^), and (**B**) carbon-to-nitrogen ratio (C:N) tended to increase with insect density, further diluting the availability of nitrogen. Shading indicates 95% confidence intervals for the regression lines.

Total vegetation biomass did not vary significantly with Mormon cricket (*p* = 0.76) or grasshopper density (*p* = 0.86), but the biomass of grasses was significantly greater than that of forbs, overall (F_1,20_ = 9.0, *p* = 0.0070). On average, grasses amounted to 21.1 g and forbs to 9.8 g of biomass in each cage. Forb mass tended to decline with the biomass of grasses (R^2^ = 0.39, F_1,8_ = 5.15, *p* = 0.053). Percent nitrogen did not change significantly with grasshopper or Mormon cricket density (*p* = 0.19 and *p* = 0.10, respectively), but it tended to decline with total insect density (*p* = 0.062, Figure 3). As a result, C:N tended to increase with total insect density (R^2^ = 0.18, F_1,18_ = 3.97, *p* = 0.062).

## 4. Discussion

Grasshoppers are a potential source of protein for Mormon crickets on U.S. western rangelands. We predicted that a decrease in grasshopper availability would result in Mormon crickets that were protein deprived. This prediction was supported by the intake diet assay, which showed that Mormon cricket preference for a protein diet increased as the grasshopper density in which they were caged decreased. We also observed Mormon crickets eating grasshoppers, including one that seized a live grasshopper on a cage wall and ate it. This makes a seemingly benign relationship between Mormon crickets and grasshoppers into a predator–prey antagonism. When sufficiently abundant, Mormon crickets could serve as an important regulator of sympatric grasshopper populations. Classic predator–prey dynamics in which the predator population growth and decline lag those of the prey are theoretically possible for Mormon crickets and their grasshopper prey [17]. However, Mormon crickets may feed on other crawling prey items, such as caterpillars, beetles, and bugs, which could dampen the influence of grasshopper abundance on that of Mormon crickets in nature. Plant-feeding by Mormon crickets can further stabilize the insects’ interactions with prey species [18].

Given that grasshoppers are a source of dietary protein for Mormon crickets, we predicted that protein-deprived Mormon crickets would have lower immunocompetence. This prediction was supported by the immunity assay, which showed that Mormon crickets caged with a low grasshopper density had lower PO activity than those with greater grasshopper numbers. In naturally migrating Mormon crickets, we found that nutrient availability has important effects on migratory velocity and immunity to disease [5,12]. Greater protein consumption boosts PO activity and the ability to ward off fungal infection [13,19]. Accordingly, grasshoppers provide an immunity boost to Mormon crickets. However, it may not only be the grasshopper protein that enhances the immune system. Grasshoppers may be a source of disease for Mormon crickets, resulting in an induced immune response of the coinhabiting Mormon crickets. Even without disease transmission, repeated physical contact can cause prophylactic changes in the immune response [20], and the Mormon crickets may have responded to higher densities of grasshoppers in the cage with a prophylactic increase in PO activity, like they respond to higher density of conspecifics [21]. Nevertheless, the result of this field experiment is consistent with previous laboratory experiments and field work that indicated that the PO activity of Mormon crickets is limited by dietary protein.

This research adds to the growing field of nutritional immunity, which focuses specifically on the role of nutrition in tolerance and defense against infections [22,23]. Some insects increase protein consumption in response to immune challenges [2,24], which may compensate for proteins lost or enhance the immune response to infection [25]. The PO cascade serves in hemolymph clotting to stop loss from wounds or to isolate foreign bodies in a melanic encapsulating coat. Mormon crickets with less protein in their diet have reduced encapsulation responses in addition to lower PO titers [13].

The omnivorous diet of Mormon crickets also makes them competitors with grasshoppers for hostplants. We found that nitrogen availability from the vegetation tended to decrease with an increase in insect density. The vegetation ranged from 0.8 to 1.7% nitrogen or 4.8 to 10.2% protein, which is much less than the 60–68% protein measured in grasshoppers (dry weights, [26]). Hence, grasshoppers are concentrating the plant protein, as are Mormon crickets, which are about 58% protein (dry weight, [27]). Greater protein intake was attainable when Mormon crickets could also feed on the grasshoppers and cannibalize one another. In a broad survey of North American grasslands [28], the biomass of omnivorous katydids was proportional to plant biomass, whereas grasshopper biomass was more closely linked to plant quality. However, with increasing plant biomass, the katydids also consumed more animal protein, which the authors suggest is due to greater prey abundance and poorer plant quality [28]. Similarly, we found that predation by Mormon crickets to meet its protein requirements became more predominant when grasshoppers were more abundant and plant quality was diminished.

In the western U.S., the uptake of soil nitrogen by plants is dependent on rainfall [29]. As a result, periodic drought and episodic rainfall make high protein resources, such as seeds, flowers, and invertebrate prey, highly variable both temporally and spatially. Grasshopper abundance might be a useful indicator that co-occurring Mormon crickets have sufficient protein in their diet, and consequently, elevated PO activity and less susceptibility to entomopathogenic fungus. In contrast, regions with low grasshopper abundance might have Mormon crickets that are particularly vulnerable to fungal attack [13], which land managers might take into consideration when applying microbial control agents [30].

Here we have shown that grasshoppers can be an important source of dietary protein and serve as a superfood for Mormon crickets, with prey availability affecting Mormon cricket immunity to disease. Grasshopper abundance could influence Mormon cricket motility due to the potential role of exercise on Mormon cricket locomotory biomechanics and the known role of protein in the migratory movement of Mormon crickets [5,8]. Furthermore, the presence of predators has been shown to alter the carbon-to-nitrogen ratios of grasshoppers and nutrient flow in grassland ecosystems [31,32,33]. Future work will examine the long-term effects of grasshopper and Mormon cricket interactions on rangeland plant community composition and nutrient cycling.

Most research on the effect of predation on immunity focuses on the prey, which typically suppresses immune function with increased predator density as a part of coping with the increased stress [34,35]. Much less research has been done on the effect of prey density on the immunity of the predator [35,36]. To the best of our knowledge, our manipulation of Mormon cricket and grasshopper densities on a meadow in the Bighorn Mountains is the first experimental study to investigate the immune response of a predator with increasing prey densities in an outdoor field setting.

We focused on grasshoppers and Mormon crickets because they are commonly co-occurring pests that reach outbreak densities on western U.S. rangeland and the exact nature of their interactions was unknown, but this initial study is only a first step. Mormon cricket populations differ in aggregation, banding, and movement behaviors [37,38,39]. When aggregated into bands, Mormon crickets typically move unidirectionally across the ground in the early morning, climb vegetation to feed in the late morning, and migrate again in the late afternoon [6,9]. The flushing and capture of prey on the ground may be an added benefit of marching in the broad (1–2 km) bands, minimizing the opportunity for prey to flee the broad band front. At a comparable elevation (2440 m) in northern Colorado [7], arthropods made up as much as 37% of the Mormon cricket diet (21% dry weight, on average). Although most of the identifiable parts were from small invertebrates, such as aphids, ants, and caterpillars, the Mormon crickets were probably not banding. At the very least, gleaning prey from vegetation when the band is not on the march is another important source of protein for migrating Mormon crickets. We have shown that Mormon crickets may provide beneficial services by consuming grasshoppers and other pest species. However, any benefits of Mormon crickets will need to be weighed against the costs, including damage to crops and competition with livestock on rangeland [40].

## Figures and Tables

**Table 1:** Sample sizes for the immunity and diet assays.

Mormon Cricket × Grasshopper Density	Mormon Cricket Sex	N-Immunity	N-Intake Diet
10 × 15	female	0	1
10 × 30	male	5	5
	female	5	4
10 × 45	male	0	1
	female	0	2
20 × 15	male	5	5
	female	5	5
20 × 30	male	2	4
	female	3	5
20 × 45	male	1	5
	female	1	3

**Table 2:** Results from MANOVAs of P and C consumption by Mormon crickets on day 1 and day 2 following removal from the caged insect density treatments.

Day	Factor	F d.f.	*P*
1	Mormon cricket density	1.1 2,34	0.34
	Grasshopper density	1.3 4,68	0.27
	Sex	5.1 2,34	0.0116
	Whole Model	1.9 8,68	0.067
2	Mormon cricket density	0.0 2,34	0.96
	Grasshopper density	3.2 4,68	0.0191
	Sex	5.1 2,34	0.0118
	Whole Model	2.9 8,68	0.0083

## Data Availability

All relevant data are included as Supplementary Tables S1–S3 in the paper.

## Supplementary Materials

The following supporting information can be downloaded at: https://www.mdpi.com/article/10.3390/insects14110868/s1, Table S1: Consumption of protein and carbohydrate in each 24 h period; Table S2: Assays of Mormon cricket immunity and hemolymph protein; and Table S3: Above ground vegetation mass and elemental composition.
